# Systematic review: YouTube recommendations and problematic content

**DOI:** 10.14763/2022.1.1652

**Published:** 2022-03-31

**Authors:** Muhsin Yesilada, Stephan Lewandowsky

**Affiliations:** University of Bristol

**Keywords:** YouTube, Extremism, Online radicalisation, Recommendation algorithms, Content recommender systems

## Abstract

There has been much concern that social media, in particular YouTube, may facilitate radicalisation and polarisation of online audiences. This systematic review aimed to determine whether the YouTube recommender system facilitates pathways to problematic content such as extremist or radicalising material. The review conducted a narrative synthesis of the papers in this area. It assessed the eligibility of 1,187 studies and excluded studies using the PRISMA process for systematic reviews, leaving a final sample of 23 studies. Overall, 14 studies implicated the YouTube recommender system in facilitating problematic content pathways, seven produced mixed results, and two did not implicate the recommender system. The review's findings indicate that the YouTube recommender system could lead users to problematic content. However, due to limited access and an incomplete understanding of the YouTube recommender system, the models built by researchers might not reflect the actual mechanisms underlying the YouTube recommender system and pathways to problematic content.

## Introduction

Social media has many positive aspects, such as creating online friendships and communities (Allen et al., 2014). However, there has also been much concern about how social media can serve as a vector for problematic content, including misinformation and conspiracy theories, that may polarise or radicalise audiences with adverse consequences for society (Allcott & Gentzkow, 2017; Allcott & Gentzkow, 2018). A recent study showed that the likelihood of users aged 15-30 encountering online hate content relating to gender, ethnicity, political views, terrorism, and religion had tripled between 2013-2015 (Kaakinen, Oksanen, & Räsänen, 2018). Other studies that investigated the same age group showed that 37% of participants reported seeing extremist content on social media platforms like YouTube (Nienierza et al., 2021). This research indicates that problematic content might be increasingly accessible. Some researchers have focused on the accessibility of problematic content on YouTube, and the role its recommender system plays in facilitating extremist content pathways (O’Callaghan et al., 2013; O’Callaghan et al., 2015; Ribeiro et al., 2020). The YouTube recommender system provides users with further viewing options based on their search and personal viewing history, along with other information about the user. Since YouTube is a massive social media platform, the notion that its algorithms facilitate pathways to extremist content is concerning. These findings have raised questions about the balance between users actively seeking out problematic content, and recommender systems leading individuals towards content that they might not otherwise have encountered.

At first glance, it may appear reasonable to assume that individuals deliberately seek content, such as videos on YouTube, that they are interested in and that is consistent with their attitudes (Knobloch, Westerwick, & Meng, 2009). On that view, YouTube users seek out content they are interested in, and the content creators supply the content to fulfil a need (Munger & Phillips, 2019). However, this simplistic view of consumer choice ignores the fact that YouTube, by default, automatically plays further videos that its recommender system deems to be of interest to the user. Even when the “autoplay” feature is turned off (itself a non-trivial undertaking), users are presented with suggested videos in a sidebar. The recommender system is integrated into YouTube’ primary structure, which organises content into videos and channels. A Channel is a unique user’s space on YouTube, where other users can find their publicly available videos. YouTube provides channel recommendations as well as video recommendations. The YouTube recommender algorithms utilise the user’s activity and the video producers’ interconnect-edness to suggest, or automatically play, videos to users (Davidson et al., 2010, p. 293; Knuth, 1997). In consequence, the recommender system—as opposed to unguided user choice—is responsible for 30% of YouTube video views (Clement & Davies, 2021).

The YouTube recommender system could direct users’ attention to video content that they otherwise might not have selected (Courtois & Timmermans, 2018; Pariser, 2011). For example, a recent study showed that users could reach conspiratorial content via the recommender system from videos about fitness, firearms, gurus, and even small houses (Alfano et al., 2020). Other studies have identified “filter bubbles” within the YouTube video network (O’Callaghan et al., 2013; O’Callaghan et al., 2015; Röchert, Weitzel, & Ross, 2020). A filter bubble refers to the algorithmically-curated presentation of homogenous content to users; this content is generally in line with the users’ interest, ideas, and beliefs (Pariser, 2011). However, the idea of a filter bubble has also been challenged (Bruns, 2019). Bruns argues that there is little empirical evidence for filter bubbles or the associated concept of political 'echo chambers’, pointing to the observation that social media users tend to be exposed to a very centrist media diet that is, if anything, more diversified than that of non-users.

Concerns about the YouTube recommender system and filter bubbles are also reflected in case studies highlighting the potential negative consequences of the algorithms, such as acts of violence that were ostensibly inspired or triggered by videos with conspiratorial content. For example, a 26-year-old man from Seattle called Buckley Wolfe killed his brother with a sword because he believed that his brother was a shape-shifting alien reptile (Green, 2019). A journalist investigated this incident by analysing Buckley's “liking” behaviour on YouTube. Buckley initially liked videos predominately about martial arts and fitness. However, his “liking” behaviour eventually shifted towards alt-lite (a loosely-defined right-wing political movement that distances itself from ethnic nationalism but opposes political correctness, feminism, and Islam), conspiracy theories, and ultimately alt-right (far-right and white nationalist) content (View, 2019). These stories, although concerning, are anecdotal and do not constitute evidence that the YouTube recommender system facilitates pathways to problematic content. Thus, assessing the evidence on the YouTube recommender system and pathways to problematic content could shed light on the extent of the issue.

There is a growing body of literature that has aimed to investigate the causal effects of social media and internet access on anti-democratic views and behaviour. For example, a study conducted in Germany and Italy showed that individuals who have greater access to broadband coverage at the municipality level were more likely to vote for populist parties (Schaub & Morisi, 2020). Another study showed that anti-refugee sentiment on Facebook predicted crimes against refugees (Müller & Schwarz, 2021), and that crimes against refugees decreased during Facebook and internet outages in localised areas. A randomised experiment conducted on a US-based sample found that deactivating Facebook four weeks before the 2018 midterm elections reduced factual news knowledge and political polarisation, suggesting a causal effect of Facebook usage on political polarisation (Allcott et al., 2020). A recent systematic review to evaluate the causal and correlational data on the relationship between digital media and political factors, including trust, polarisation, and news consumption found a mixed pattern (Lorenz-Spreen et al., 2021). Some associations, for example, increases in political involvement and information consumption are expected to be good for democracy and have been documented frequently in the Global South and developing democracies. Other associations, such as diminishing political trust, populist advantages, and increased polarisation, are likely anti-democratic and more prominent in established democracies. Crucially, some of the studies reviewed by Lorenz-Spreen et al. used a variety of statistical techniques (e.g., instrumental-variable analysis) to establish causality, rather than a mere association, thus providing some support for the idea that social media can cause negative political behaviours and attitudes.

YouTube itself acknowledges the potential adverse impacts of social media and recently took steps to promote accurate information and reduce harmful content by making changes to their algorithms (“YouTube community guidelines”, n.d.). YouTube cites 4 Rs of responsibility: remove harmful content, raise authoritative voices as a means of promoting accurate information, reward trusted creators, and reduce the spread of content that brushes right up against the policy line. Between October 2020 to December 2020, YouTube reported that they removed 3.8 million videos that violated child safety rules, 1.4 million spam, misleading, and scam videos, 259,000 harmful or abusive videos, 73,000 videos that promoted violence or violent extremism (“Progress on managing harmful content”, 2021). These remedial measures indicate that YouTube is aware of the accessibility of problematic content on its platform.

A review of the existing literature on the YouTube recommender system in the context of facilitating radicalisation could shed light on the success of the platform’s measures. YouTube’s role in facilitating access to problematic content via the recommender system is still much debated. As a means of filling this knowledge gap, we present a systematic review of the existing evidence for whether the YouTube recommender system facilitates pathways to problematic content. To our knowledge, this is the first systematic review of its kind. We define the facilitation of problematic content as the process whereby the YouTube recommender systems recommends problematic content from a channel that has not posted problematic content, or further problematic content from a video/channel that itself contained problematic content. We further define problematic content as content that violates the community guidelines and policies set out by YouTube as of 2020. YouTube regards sensitive content such as indecent videos of children, nudity and sexual content, suicide, and self-injury as a violation of community guidelines and policies (“YouTube community guidelines”, n.d.). YouTube also regards content containing violent or dangerous content such as hate speech, extremist content, content from violent criminal organisations, and misinformation as a violation of community guidelines and policies (“YouTube community guidelines”, n.d.).

Our review utilised key terms associated with these kinds of problematic content during the search for relevant studies (see Table 1 for a definition of these terms). The review included studies if they investigated at least one of those types of problematic content or if we deemed the content a violation of YouTube guidelines and policies. Each study included in the review had a classification method to identify problematic content and their methods are outlined in the methodology table (see Supplementary Materials S2).

## Method

**Figure 1:**
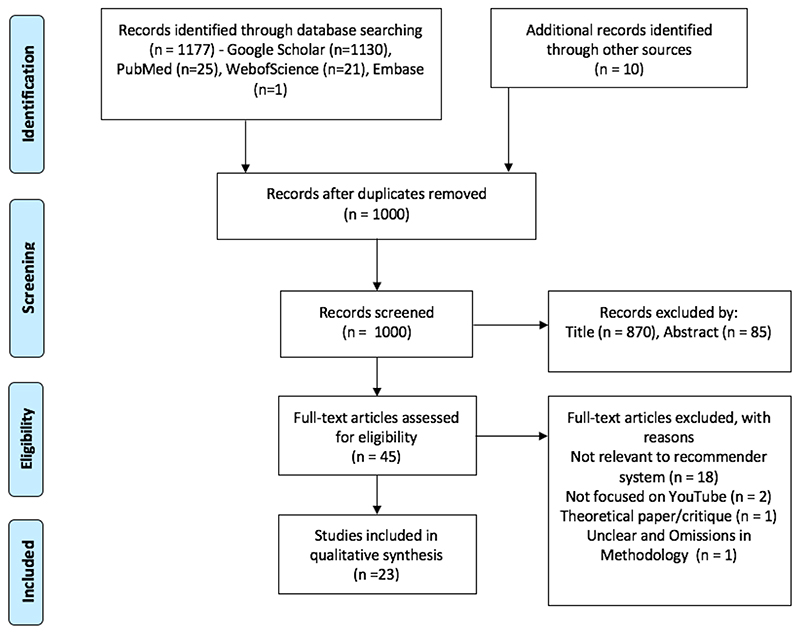
Exclusion process – PRISMA

We searched and extracted studies from Google Scholar, Embase, Web of Science, and PubMed for relevant studies using Boolean operators and search terms (see Table 2), resulting in a database of 1,187 studies. The studies were then systematically filtered in line with the eligibility and exclusion criteria (see below). The review followed the guidelines set out by PRISMA for excluding studies in systematic reviews. The PRISMA (Moher, Liberati, Tetzlaff, & Altman; The PRISMA Group, 2009b) flowchart (Figure 1) summarises the filtering process to exclude studies that did not fulfil the eligibility criteria. Studies were first excluded by title, and then abstract, and then the remaining studies were excluded by reading the full text. The meta-data, methodology, classifications, and results of the remaining studies were then put into four tables representing, respectively, meta-data (Supplementary Materials S1), methodology (Supplementary Materials S2), results (Supplementary Materials S3), and the classification of problematic content (Supplementary Materials S4). At each point of the exclusion process, the studies were assessed against the eligibility criteria (see below). If the authors determined that a study failed to meet the eligibility criteria, they were excluded.

### Eligibility criteria

A set of eligibility criteria was put together to identify suitable studies. The research quality of the candidate papers was assumed to be equal. Studies had to meet the following criteria for inclusion in this review:

- The studies must explicitly investigate YouTube, or at least include YouTube in the analysis. Studies that investigated recommender systems for other platforms, or other social media platforms in general (such as Twitter and Facebook) were excluded.
- The studies must explicitly focus on the YouTube Recommender System. The study can focus on either channel or video recommendations. Studies that aimed to create classifiers of problematic content were included if they also investigated the recommender system.
- The studies must explicitly focus on whether the YouTube Recommender System facilitates pathways to problematic content.
- The studies must be published in a peer-reviewed journal or as a preprint, and the full text must be available in English. Dissertations were excluded irrespective of whether they were written in English.
- Studies must focus on problematic content, including but not limited to extremist messages, extreme right, hate speech, extremism, alt-right, extreme left, Islamophobia, Islamist extremism, radicalisation, conspiracy theories, misinformation, and disinformation (See Table 1 for definitions). The studies at times have differing definitions of problematic content. Some papers use a particular type of problematic content as a proxy for another type of problematic content; for example, conspiratorial content is sometimes used as a proxy for extremist content (see Table S4).
- The study must provide adequate coverage of the methodology and results section. The authors assessed whether the studies provided a clear overview of how they collected their data and their video classification procedure. The results had to clearly set out the findings supported by quantitative measurements.

### Search strategy

Google Scholar, Web of Science, Medline, PubMed, Embase were searched between November and December 2020. This combination of databases has been shown to perform best at achieving efficient and adequate coverage of studies (Bramer, Rethlefsen, Kleijnen, & Franco, 2017). Each database was searched using a set of Boolean operators and truncations (see Table 2). Studies that were not detected by the search terms but were sent to the researchers by colleagues throughout the investigation were also included. We contacted authors that we were aware of who have researched relevant areas to send over papers that they felt were relevant.

### Data extraction

Duplicate studies were deleted from the initial set of 1,187 studies returned by the keyword searches and from other sources (such as studies shared with the researchers directly). The set of unique studies was then subjected to a three-stage screening procedure (See Figure 1) using the criteria outlined earlier. At stage 1, the studies were screened by title. At stage 2, the remaining papers were then screened by abstract. Finally, at stage 3, the papers were screened by full text, and the remaining studies were included in the review.

The authors then created four tables to describe the studies included in the review. The first table described the meta-data for each study, namely title, corresponding author, number of citations, and publication date (see Supplementary Materials S1). The second table described the methods used by each of the studies and included problematic content type, number of seed videos/channels (a seed video/channel describes a starting point for the data collection, recommended videos/channels are collected from these starting points), video classification method, search queries, and the data analysis method (see Supplementary Materials S2). The third table described each study's results and whether the results implicated the YouTube recommender system in facilitating pathways to problematic content (see Supplementary Materials S3). The fourth table describes the types of problematic content investigated in each study and whether each type of problematic content was the primary problematic content or a proxy of the target problematic content. The table also describes if the included studies found evidence to implicate the recommender system in facilitating problematic content pathways or not (see Supplementary Materials S4).

### Data analysis

A meta-analysis was not conducted due to the varied study designs and outcome measures. Instead, a narrative synthesis was produced. A narrative synthesis verbally summarises, explains, and compares the results and methodology within and between studies (Popay et al., 1995). The narrative synthesis helped to understand the extent to which the studies implicate the YouTube recommender system in facilitating pathways to extremist and problematic content. Each study is described below, followed by a comparative analysis.

## Results

### Metadata of studies

The upload or publication dates for the final set of 23 studies ranged from 2013-2021. Two studies focused on anti-vaccine content, three studies on conspiratorial content, seven studies on extremist content, two studies on radicalising content, four studies on unsafe content for children, one on incel-related content (the term incel describes a community of men who feel that they are treated unfairly by society and women and can display extreme misogyny), three on pseudo-scientific content, and one on racism. The extremist content and radicalising content categories are similar but subtly different. For example, although the videos in the radicalising content category also investigate extremist content, they focus on the possibility of a radicalisation process through the recommender system. That is, they investigate whether users are exposed to increasingly extremist content via the recommender system. It should be noted that this is not to suggest that individuals become more radical as they are exposed to more radical content. Instead, the authors of these papers investigate exposure to increasingly radical content irrespective of its consequences. The videos in the extremist content category focus mostly on analysing the formation of filter bubbles, whereas the radicalising content videos investigate pathways to extremist content from innocuous or apolitical starting points.

Some studies included other types of problematic content as a proxy for the type of problematic content they intended to investigate (see Supplementary Materials S4 for the problematic content classification table). For example, a study investigating extremist content classified videos that contained conspiratorial content as extremist (Röchert, Weitzel, & Ross, 2020). Four of the 23 studies accounted for user personalisation in their methodology. User personalisation is the process by which YouTube provides recommendations based on factors such as users’ interaction with other Google products and their personal search history. These studies created accounts and built search and watch histories to account for user personalisation (see Supplementary Materials S2 for methodologies).

The mean number of Google scholar citations across the 23 studies was 20.36 (SD=32.82, range = 0 - 133). The studies collectively analysed 1,347,949 YouTube videos/channels (see Table S1 for meta-data information on the included studies).

### Recommender systems and pathways to problematic content

Overall, 14 studies implicated the YouTube recommender system in facilitating problematic content pathways (Alfano et al., 2020; AVAAZ Report, 2020; Chen et al., 2021; Hussein et al., 2020; Matamoros-Fernández, 2017; O’Callaghan et al., 2013; O’Callaghan et al., 2015; Papadamou et al., 2020; Papadamou et al., 2020; Röchert, Weitzel, & Ross, 2020; Schmitt et al., 2018; Song & Gruzd, 2017; Spinelli & Crovella, 2020). Two studies did not implicate the YouTube recommender system in facilitating problematic content pathways (Hosseinmardi et al., 2020; Ledwich & Zaitsev, 2019). Finally, seven studies produced mixed results (Abul-Fottouh, Song, & Gruzd, 2020; Faddoul, Chaslot, & Farid, 2020; Kaiser & Rauchfleisch, 2019; Kaushal et al., 2016; Papadamou et al., 2020; Ribeiro et al., 2020).

### Narrative synthesis

The following section produces an analysis of the included studies in subsections depending on the content focus of the studies: conspiratorial content, anti-vaccine content, extremist content, radicalised content, content unsafe for children, “incel”-related content, pseudoscientific content, and racist content. The studies had differing classification processes for identifying and labelling problematic content. The methods table in Supplementary Materials S2 outlines how each study classified videos and channels as problematic content (for example, extremist). The methods table also provides further details about the data analysis procedure used by the studies.

#### Conspiratorial content

Three studies investigated conspiratorial content (Alfano et al., 2020; Hussein et al., 2020). The study conducted by Alfano et al. (2020) used a web crawler to simulate a user consuming content via the recommender. The aim of the study was to determine if the YouTube recommender amplified and facilitated access to conspiracy theories. The study showed that individuals could reach conspiratorial content via the recommender system from reasonably innocuous starting points, such as videos about martial arts and tiny houses. The study by Hussein et al. (2020) used a different methodology, and created bots and staged google accounts. The bots and staged accounts that watched conspiratorial content received recommendations for further conspiratorial content. The findings support the notion of a filter bubble effect, as suggested by Pariser (2011). By contrast, the study by Faddoul et al. (2020) found mixed results. The authors ran a longitudinal analysis to investigate the frequency with which the YouTube algorithms recommend conspiratorial content. Throughout the longitudinal analysis, the number of conspiratorial content recommendations made by the YouTube recommender system decreased. However, Faddoul et al. (2020) also presented evidence that the recommender system can recommend conspiratorial content if the user watches conspiratorial content as a starting point, supporting the notion of a filter bubble effect.

Other studies included conspiratorial content in their investigations albeit as a proxy for another type of problematic content. Those studies are thus described in other sub-sections. For example, Ledwich and Zaitsev (2019) included conspiratorial content as a proxy of radicalising content, so this study is discussed in the radicalising content section.

#### Anti-vaccination content

Two studies investigated anti-vaccination content (Abul-Fottouh, Song, & Gruzd, 2020; Song & Gruzd, 2017). Song and Gruzd (2017) conducted a social network analysis to determine the importance of anti-vaccination content in a network of vaccine-related videos. The results suggested that watching anti-vaccine content facilitated pathways via the recommender system to more anti-vaccine content. The most recent study conducted by Abul-Fottouh, Song, and Gruzd (2020) also used social network analysis to determine the prominence of anti-vaccine and pro-vaccine videos. The results showed that YouTube recommended more pro-vaccine videos than anti-vaccine videos. Over time, these differing results could reflect YouTube’s recent efforts to demonetise harmful content, reduce harmful misinformation, and their changes to the recommendation algorithm (YouTube, 2019). For example, YouTube is currently taking further steps to remove anti-vaccine content from the platform, perhaps as a consequence of the COVID-19 pandemic (Reuters, 2021).

A study conducted by Papadamou et al. (2020) also included anti-vaccine content. However, because they classified anti-vaccine content as a proxy of pseudoscientific content, the study is described in the next section.

#### Pseudoscientific content

The study by Papadamou et al. (2020) obtained mixed results. For example, users were more likely to encounter pseudoscientific videos on the platform’s search results page than through the recommender system or the homepage. A user was more likely to encounter pseudoscientific content when searching for anti-vaccine, anti-mask, and flat earth content than encountering it via the recommender system. The study also found that the recommender system was unlikely to suggest COVID-19 pseudoscientific content in comparison to other types of pseudoscientific content. This finding may indicate that YouTube’s recent efforts to minimise the spread of problematic COVID-19 content were successful (YouTube, n.d.). In contrast, a study conducted by Spinelli and Crovella (2020) simulated user behaviour (i.e., used an algorithm to simulate a user watching content on YouTube and then randomly selecting a recommended video to watch next) and found that the recommender system facilitated pathways to extreme and unscientific content from reliable information sources. Finally, a social network analysis included in a recent report found that 16% of the top 100 videos under the “up next” option related to the search term “global warming” contained misinformation about climate change (AVAAZ, 2020). The findings showed that watching climate change misinformation videos often led to further recommendations of climate change misinformation videos (AVAAZ, 2020).

#### Content unsafe for children

Four studies investigated the YouTube recommender system in the context of facilitating pathways to content that is unsafe for children, such as video containing sexually suggestive content – which is a violation of YouTubes community guidelines and thus included in our analysis. The study conducted by Papadamou et al. (2020) found that children are likely to encounter disturbing content via the recommender system from videos classified as appropriate for children aged one to five. However, the studies conducted by Kaiser and Rauchfleisch (2019) and Kaushal et al. (2016) found mixed results. For example, Kaiser and Rauchfleisch (2019) created a directed graph of nodes (videos) and edges (video recommendations). By structuring the recommender system into a graph, the authors identified potential pathways (via the edges) to inappropriate content. They found that the most inappropriate content was ten clicks away via the recommender system.

Kaushal et al. (2016) found that safe-to-unsafe content transitions are rare, but un-safe-to-unsafe transitions are common. The study conducted by Stöcker and Preuss (2020) simulated users consuming content via the recommender system. The authors argue that contextually inappropriate content (in this context for children) is not the recommender system's goal, and instead, they argue it is collateral damage. That is, the system sometimes leads to the promotion of problematic content to a broader audience. The authors particularly implicate the autoplay feature in recommending inappropriate content. Overall, the four papers collectively agree that unsafe content is accessible. However, there is disagreement on the likelihood of accessing inappropriate content via the recommender system.

#### Incel-related content

One study investigated pathways via the recommender system to incel-related content. Incel is short for “involuntarily celibate” and describes a male individual who blames his inability to find a romantic partner on society being designed to benefit attractive people. At the extreme end of these communities, incels see radical, extreme and violent action as the solution to the perceived unfairness. Violence is often targeted towards women (Cook, 2018). Incel ideology is associated with misogyny, alt-right ideology, and anti-feminist views. Incel ideology has been implicated in mass murders and violent offences (S.P.L. Center, 2019). The study conducted by Papadamou et al. (2020) implicated the recommender system in facilitating incel-related content pathways. A random walk analysis determined an 18.8% probability that users could see at least one incel-related video from a benign starting point within five steps through the recommender system. However, it is difficult to draw firm conclusions from the results of a single study, and further examinations of incel-related content may be necessary to understand this content area.

#### Extremist content

Seven studies investigated the YouTube recommender system in the context of facilitating pathways to extremist content (Chen et al., 2021; Hosseinmardi et al., 2020; O’Callaghan et al., 2013; O’Callaghan et al., 2015; Kaiser & Rauchfleisch, 2020; Röchert, Weitzel, & Ross, 2020; Schmitt et al., 2018). Six studies implicated the recommender system in facilitating pathways to extremist content. Five of these studies establish a link between filter bubbles and the recommender system (Chen et al., 2021; O’Callaghan et al., 2013; O’Callaghan et al., 2015; Kaiser & Rauchfleisch, 2020; Röchert, Weitzel, & Ross, 2020). Schmitt et al. (2018) found that extremist content is at times closely or directly related to counter-message content (i.e., anti-extremist material provided by public-interest organisations or the government) via the recommender system. For example, Schmitt et al. found that users could encounter extremist content within two-clicks through the recommender system from the counter-message content. This finding highlights the challenges faced by counter messages; users might be placed into closer proximity to extremist content through a counter message than they might have been otherwise.

A study conducted by Chen et al. (2021) used a browser plug-in to track participants’ watch history and YouTube algorithmic recommendations. The results showed that 9.2% of participants viewed an extremist channel video, and 22.1% viewed a video from an Alternative Influence Network channel. The Alternative Influence Network (AIN) describes a group of channels ranging from white nationalists to those who identify as conservative and libertarian thinkers (Lewis, 2018). Lewis argues that they are united by their general rejection of feminism, social justice, or left-wing political ideology. When participants watched these videos, they were more likely to be recommended similar videos. The study by Chen et al. (2021) demonstrates that the YouTube recommender system could facilitate access to more problematic content after a user consumed an initial video. Interestingly, 90% of views for both AIN and extremist videos came from participants who scored highly in racial resentment, indicating the importance of prior attitudes to accessing problematic content.

Another study used panellist data of participants who had logged onto YouTube at least once. The methodology aimed to identify consecutive page views by a user and assigned users to communities of highly related videos. To determine overall trends to far-right content, overall changes in total user consumption associated with each of the content communities was investigated. The analysis demonstrated growing far-right echo chambers, however, no evidence to support the notion that they are caused by the recommendation algorithm (Hosseinmardi et al., 2020).

#### Radicalising content

Two studies investigated the link between the recommender system and pathways to radicalising content (Ledwich & Zaitsev, 2019; Ribeiro et al., 2020). The study conducted by Ledwich and Zaitsev (2019) created a protocol to estimate the number of times a recommendation was displayed to a user and concluded that the recommender system does not facilitate pathways to radicalising or extremist content. By contrast, the study conducted by Ribeiro et al. (2020) found pathways via the channel recommender system from alt-lite (a loosely defined right-wing group) and Intellectual Dark Web Content (IDW; a group of commentators who discuss controversial topics but do not necessarily endorse extremist ideology) to alt-right content, but not via recommended videos. However, Ribeiro et al. also find alt-right content from recommended channels, but not from recommended videos. Thus, there is a difference in findings between the two studies. Ledwich and Zaitsev (2019) suggest that content creators are responsible for the accessibility of problematic content rather than YouTube itself. They agree with Munger and Phillips’ (2019) view that YouTube can be conceptualised as a supply and demand framework. This notion suggests that users seek out content that they are actively interested in viewing rather than passively being led to problematic content from innocuous starting points.

#### Racist content

One study aimed to analyse racism from an Australian-based controversy on social media platforms (Matamoros-Fernández, 2017). The controversy was based on racism directed at an Australian football player called Adam Goodes. According to the results, the recommender system facilitated controversial humour about the Adam Goodes racism topic, and videos by public figures who have shared racist remarks about Aboriginal people. Since this study aimed to investigate a specific racist incident, more research is required to determine the extent to which the YouTube recommender system facilitates pathways to further racist content.

Other studies have investigated racist content but classified racist content as a proxy of another type of problematic content and are thus described in the other subsections. For example, Ledwich and Zaitsev (2019) investigated radicalising content and labelled certain problematic videos as white identitarian (groups or individuals who believe that “whites” are the superior race) and anti-whiteness. These labels include essentialist concepts of racial differences and strictly frame current events as racial oppression. Similarly, the study conducted by O’Callaghan et al. (2015) included white nationalist content in their investigation of extremist content. According to their topic modelling approach, racism was a proxy topic of videos fitting into the white nationalist political party category.

## Discussion

### Summary

This systematic review aimed to examine whether the YouTube recommender system facilitates pathways to problematic content. Overall, most of the 23 included studies found evidence to implicate YouTube in facilitating pathways to problematic content or found mixed results. Nine of the studies demonstrated evidence to support the notion of a filter bubble effect (AVAAZ, 2020; Chen et al., 2021; O’Callaghan et al., 2013; Faddoul et al., 2020; Hussein et al., 2020; O’Callaghan et al., 2015; Kaiser & Rauchfleisch, 2020; Röchert, Weitzel, & Ross, 2020; Song & Gruzd, 2017). Only two investigations did not implicate the YouTube recommender system in facilitating paths to problematic content (Hosseinmardi et al., 2020; Ledwich & Zaitsev, 2019).

### Implications

The rise of social media platforms presents new challenges to users. Individuals should be cautious about the content they consume and how they behave on social media platforms such as YouTube. Alfano et al. (2020) call for epistemic vigilance as a means of conferring resistance to conspiratorial content. For example, they argue that deactivating autoplay could encourage users to think about what they would like to watch next rather than leaving the decisions solely to the recommender system.

Studies such as Schmitt et al. (2018) demonstrate that counter-message videos could counter-productively facilitate extremist content pathways. Changes to the recommender system could maximise the benefit of introducing counter-messaging. For example, these counter-messages could be placed as ads on videos identified as problematic. These ads could implement principles from inoculation theory to increase users’ resistance to persuasive problematic content. Inoculation strategies equip individuals with the ability to critically assess and refute potential misinformation by revealing the logical fallacies or rhetorical deficiencies in misleading communications before people are exposed to it (Cook et al., 2017). A recent study demonstrated that presenting participants with a brief inoculation video increases their resistance to Islamophobic and Islamist extremist disinformation (Lewandowsky & Yesilada, 2020). Participants in the inoculation condition were less likely to want to share the disinformation, perceived the disinformation as less reliable, agreed less with the misinformation, and indicated less support for the misinformation. Policymakers are increasingly entertaining social media regulation, and this rollout would provide one possible target for policy intervention without incurring the risk of censorship (Lewandowsky & Yesilada, 2020).

### Common limitations of these studies and future directions

For the most part, the studies reviewed here did not consider user personalisation (see Supplementary Materials S2 for an outline of each study’s methodology). The recommendations were often based on seed videos and did not account for common factors like users’ interaction with other Google products and their personal search history. Many papers in this area use random walk algorithms to determine the probability of encountering problematic content via the recommender system and thus do not account for user personalisation. Only two of the most recent studies in this area utilised a plug-in to follow real YouTube users and their recommendations (Chen et al., 2021; Hosseinmardi et al., 2020). Plug-ins could lead to a deeper understanding of how user characteristics impact watch choices. Because plug-ins are based on real user recommendations, they can provide deeper insights into pathways to problematic content. Munger and Phillips (2020) note the supply and demand concept of user watch choice. These studies could provide insight into whether the YouTube recommender system facilitates pathways to problematic content irrespective of the user's characteristics and preferences. Future studies should follow-up on this work and focus on how user personalisation impacts the recommendations provided by YouTube. These future studies could provide useful insights into the YouTube recommender system and problematic content pathways.

However, these panel studies are also not without their limitations. As is the case for one study, the panel-based technique benefits by directly monitoring consumption. It also allows the researchers to view movies that were recommended but not chosen, according to Hosseinmardi et al. (2020). As a result, a mix of panel and platform data would be required to reconstruct consumers' decision-making processes fully. However, this is difficult because YouTube does not fully disclose its algorithms nor provide users' watch choices. Although, the plug-in methodology employed by Chen et al. (2020) seems like an effective strategy in recording users' watch choices.

Thus, it is challenging to characterise the YouTube recommender system accurately in a single headline. Most studies work from a limited list of search seeds and only provide a snapshot of the whole YouTube network. The authors of these studies often recognise the methodological limitations. For example, Ribeiro et al. (2020) point out that their methodology can only provide a small snapshot of the recommender system, and to fully replicate and analyse the recommender system is beyond the capabilities of researchers external to YouTube. Assessing the validity of concepts such as the ‘filter bubble effect’ therefore remains a challenging task. Significant progress could be made if YouTube worked closely with researchers to determine the recommender system’s role in facilitating pathways to problematic content.

The included studies do not explain the *why* behind these pathways to inappropriate content, nor do they elucidate the consequences of embarking down these paths. A study by Alfano et al. (2020) that investigated the YouTube recommender system and pathway to conspiratorial content highlights this issue in their methodology. They explain that real users might not follow the path they describe, nor do they provide evidence of how many of them might do so. As a result, it is as yet unknown whether the recommender system results in transformational experiences of the kind that could cause a significant shift (for better or worse) in one's epistemic perspective. It is possible that YouTube videos fill in the specifics for people already inclined to believe conspiracy theories rather than that YouTube converts non-conspiracy theorists to conspiracy theorists. Against this possibility, there is a growing body of literature that has determined real-world adverse causal effects of social media usage on polarisation and violence (Allcott et al., 2020; Müller & Schwarz, 2018; Schaub & Morisi, 2020). Future research should clear up these knowledge gaps by exploring the consequences of exposure to inappropriate content via the YouTube recommender system.

## Conclusions

Overall, the results suggest that the YouTube recommender system can facilitate pathways to problematic content. Although further research and collaboration with social media companies is needed to understand the underlying mechanisms in which this process occurs and its consequence, the review sums up the current understanding of the issue. The results could call for further actions to be taken by YouTube to steer people away from problematic content. YouTube could work with external researchers to provide greater insight into the recommender system as a means of auditing its user safety. Currently, researchers are aware of the limitations of their methodologies due to restricted access, and greater access could lead to more rigorous research practices.

## Supplementary Material

Supplementary Material

## Figures and Tables

**Table 1:** Search term definitions

SEARCH TERM	DEFINITION
Extreme right	A right-wing political movement that is more extreme than traditional conservative ideology
Extreme left	A left-wing political movement that is more extreme than traditional liberal ideology
Hate Speech	An expression of hate based on race, religion, or sexual orientation (Cambridge Dictionary, n.d.)
Extremism	The quality or state of being extreme, often used in political and religious settings (The Free Dictionary, n.d.)
Alt-right	A far-right, white nationalist movement
Islamophobia	The fear, hatred, and prejudice against Muslims or Islam (Cambridge Dictionary, n.d.)
Islamist extremism	Extremism associated with the religion of Islam
Extremist messages	Messages that spread an extremist ideology
Misinformation	False information, regardless of its intent to deceive (Dictionary, n.d.)
Disinformation	False information, that is actively trying to deceive (Dictionary, n.d.)
Conspiracy Theories	Explains a situation that invokes a conspiracy by sinister and powerful groups or individuals, without the presence of evidence (Brotherton, Robert; French, Christopher Pickering, & Alan, 2013)
Radicalisation	The adoption of increasingly radical views that move against the norms and values of society.

**Table 2:** Search keys

KEY TERMS	SEARCH TERMS
Recommender system key terms	(Information network analysis OR Recommender systems OR Recommender system OR Recommendation algorithm)
AND	
YouTube content key terms	(YouTube content analysis OR YouTube)
AND	
Type of content key terms	(Counter-messages OR Extremist messages OR Extreme right OR Hate Speech OR Hate OR Extremism OR Alt-right OR Extreme left OR Islamophobia OR Islamist OR Radicalisation OR Radicalisation OR Conspiracy Theory OR Misinformation OR Disinformation)

## References

[R1] Abul-Fottouh D, Song MY, Gruzd A (2020). Examining algorithmic biases in YouTube’s recommendations of vaccine videos. International Journal of Medical Informatics.

[R2] Alfano M, Fard AE, Carter JA, Clutton P, Klein C (2021). Technologically scaffolded atypical cognition: The case of YouTube’s recommender system. Synthese.

[R3] Allcott H, Braghieri L, Eichmeyer S, Gentzkow M (2020). The welfare effects of social media. American Economic Review.

[R4] Allcott H, Gentzkow M (2017). Social media and fake news in the 2016 election. Journal of Economic Perspectives.

[R5] Allcott H, Gentzkow M, Yu C (2019). Trends in the diffusion of misinformation on social media. Research Politics.

[R6] Allen KA, Ryan T, Gray DL, McInerney DM, Waters L (2014). Social media use and social connectedness in adolescents: The positives and the potential pitfalls. Australian Educational Developmental Psychologist.

[R7] Avaaz (2020). Why is YouTube broadcasting climate misinformation to millions? [Report]. Avaaz.

[R8] Brotherton R, French CC, Pickering AD (2013). Measuring belief in conspiracy theories: The generic conspiracist beliefs scale. Frontiers in Psychology.

[R9] Cambridge Dictionary (2021a). Hate speech.

[R10] Cambridge Dictionary (2021b). Islamaphobia.

[R11] Chen A, Nyhan B, Reifler J, Robertson R, Wilson C (2021). Exposure to Alternative Extremist Content on YouTube [Report]. Anti-Defamation League.

[R12] Clement J, Davies P (2020). Impact of recommendation engine on vdeo-sharing platform-YouTube.

[R13] Cook J (2018). A toxic ‘brotherhood’: Inside incels’ dark online world. Huffington Post.

[R14] Courtois C, Timmermans E (2018). Cracking the Tinder Code: An Experience Sampling Approach to the Dynamics and Impact of Platform Governing Algorithms. Journal of Computer-Mediated Communication.

[R15] Davidson J, Livingston B, Sampath D, Liebald B, Liu J, Nandy P, Van Vleet T, Gargi U, Gupta S, He Y, Lambert M (2010). The YouTube video recommendation system.

[R16] (2021). Definition of disinformation.

[R17] (2021). Definition of misinformation.

[R18] Faddoul M, Chaslot G, Farid H (2020). A longitudinal analysis of YouTube’s promotion of conspiracy videos.

[R19] Google (2021). COVID-19 medical misinformation policy. YouTube Help.

[R20] Green SJ (2019). God told me he was a lizard’: Seattle man accused of killing his brother with a sword. The Seattle Times.

[R21] Hosseinmardi H, Ghasemian A, Clauset A, Rothschild DM, Mobius M, Watts DJ (2020). Evaluating the scale, growth, and origins of right-wing echo chambers on YouTube. ArXiv.

[R22] Kaakinen M, Oksanen A, Räsänen P (2018). Did the risk of exposure to online hate increase after the November 2015 Paris attacks? A group relations approach. Computers in Human Behavior.

[R23] Kaiser J, Rauchfleisch A (2019). The implications of venturing down the rabbit hole. Internet Policy Review.

[R24] Kaushal R, Saha S, Bajaj P, Kumaraguru P (2016). KidsTube: Detection, characterization and analysis of child unsafe content promoters on YouTube.

[R25] Knobloch-Westerwick S, Meng J (2009). Looking the other way: Selective exposure to attitude-consistent and counterattitudinal political information. Communication Research.

[R26] Knuth DE (1997). The Art of Computer Programming: Fundamental Algorithms.

[R27] Ledwich M, Zaitsev A (2019). Algorithmic extremism: Examining YouTube’s rabbit hole of radicalization.

[R28] Lewandowsky S, Yesilada M (2021). Inoculating against the spread of Islamophobic and radical-Islamist disinformation. Cognitive Research: Principles and Implications.

[R29] Lewis R (2018). Alternative influence: Broadcasting the reactionary right on YouTube [Report]. Data Society.

[R30] Littler M (2019). Exploring radicalisation and extremism online: An experimental study [Report].

[R31] Matamoros-Fernández A (2017). Platformed racism: The mediation and circulation of an Australian race-based controversy on Twitter, Facebook and YouTube. Information, Communication Society.

[R32] Müller K, Schwarz C (2021). Fanning the Flames of Hate: Social Media and Hate Crime. Journal of the European Economic Association.

[R33] Nienierza A, Reinemann C, Fawzi N, Riesmeyer C, Neumann K (2021). Too dark to see? Explaining adolescents’ contact with online extremism and their ability to recognize it. Information, Communication Society.

[R34] O’Callaghan D, Greene D, Conway M, Carthy J, Cunningham P (2013). The extreme right filter bubble.

[R35] O’Callaghan D, Greene D, Conway M, Carthy J, Cunningham P (2015). Down the (white) rabbit hole: The extreme right and online recommender systems. Social Science Computer Review.

[R36] Papadamou K, Papasavva A, Zannettou S, Blackburn J, Kourtellis N, Leontiadis I, Sirivianos M (2020). Disturbed YouTube for children: Characterizing and detecting inappropriate videos targeting young children.

[R37] Papadamou K, Zannettou S, Blackburn J, De Cristofaro E, Stringhini G, Sirivianos M (2020a). “How over is it?” Understanding the Incel Community on YouTube.

[R38] Papadamou K, Zannettou S, Blackburn J, De Cristofaro E, Stringhini G, Sirivianos M (2020b). “It is just a flu”: Assessing the Effect of Watch History on YouTube’s Pseudoscientific Video Recommendations.

[R39] Pariser E (2011). The Filter Bubble: What The Internet Is Hiding From You.

[R40] Popay J, Roberts H, Sowden A, Petticrew M, Arai L, Rodgers M, Britten N, Roen K, Duffy S (2006). Guidance on the conduct of narrative synthesis in systematic reviews: A product from the ESRC Methods Programme.

[R41] (2021). Progress on managing harmful content. YouTube.

[R42] Reuters (2021). YouTube blocks all anti-vaccine content. Reuters.

[R43] Ribeiro MH, Ottoni R, West R, Almeida VAF, Meira W (2019). Auditing Radicalization Pathways on YouTube.

[R44] Röchert D, Weitzel M, Ross B (2020). The homogeneity of right-wing populist and radical content in YouTube recommendations.

[R45] Schaub M, Morisi D (2020). Voter mobilisation in the echo chamber: Broadband internet and the rise of populism in Europe. European Journal of Political Research.

[R46] Schmitt JB, Rieger D, Rutkowski O, Ernst J (2018). Counter-messages as prevention or promotion of extremism?! The potential role of youtube: Recommendation algorithms. Journal of Communication.

[R47] Song MY-J, Gruzd A (2017). Examining Sentiments and Popularity of Pro-and Anti-Vaccination Videos on YouTube.

[R48] S.P.L. Center (2019). Male Supremacy.

[R49] The YouTube Team (2021). The Four Rs Of Responsibility, Part 2: Raising Authoritative Content And Reducing Borderline Content And Harmful Misinformation. YouTube Official Blog.

[R50] TheFreeDictionary.com (2021). Extremism. TheFreeDictionary.

[R51] View T (2019). I Was Looking At The “Likes” On The Youtube Page Of Buckey Wolfe, The Qanon Follower Who Is Accused Of Killing His Brother. Wolfe Also Told [...]”.

[R52] YouTube community guidelines and policies—How YouTube Works. YouTube.

