# Supplementary material for: Systematic review: YouTube recommendations and problematic content
Source: Internet Policy Rev. Author manuscript; Available in PMC 2022 Dec 1. (PMC7613872; doi:10.14763/2022.1.1652)
Supplement: Supplementary Material [file EMS156628-supplement-Supplementary_Material.pdf]

Online Supplementary Materials for: A Systematic Review: The YouTube Recommender System and Pathways to Problematic Content

Online Supplementary Materials for: A Systematic Review: The YouTube Recommender System and Pathways to Problematic Content

Supplementary Materials S1: Meta-Data Table

Title	Citation	Aim	Upload Year	Google Scholar Citations
Technologically scaffolded atypical cognition: the case of YouTube's recommender system	Alfano, M., Fard, A. E., Carter, J. A., Clutton, P., & Klein, C. (2020). Technologically scaffolded atypical cognition: The case of YouTube, recommender system. Synthese, 1-24.	This study aimed to establish whether and to what extent the recommender system promotes conspiratorial content.	2020	5
Examining algorithmic biases in YouTube's recommendations of vaccine videos	Abul-Fottouh, D., Song, M. Y., & Gruzd, A. (2020). Examining algorithmic biases in YouTube, recommendations of vaccine videos. International Journal of Medical Informatics, 140, 104175.	This research investigates the YouTube recommender system in the context of vaccination-related videos.	2020	1
Counter-messages as Prevention or Promotion of Extremism?! The Potential Role of YouTube: Recommendation Algorithms	Schmitt, J. B., Rieger, D., Rutkowski, O., & Ernst, J. (2018). Counter-messages as prevention or promotion of extremism?! The potential role of YouTube: recommendation algorithms. Journal of communication, 68(4), 780-808.	This study investigated the extent to which the YouTube recommender system facilitates pathways between counter- and extremist messages	2018	34
Algorithmic Extremism: Examining YouTube, Rabbit Hole of Radicalization	Ledwich, M., & Zaitsev, A. (2019). Algorithmic extremism: Examining YouTube's rabbit hole of radicalization. arXiv preprint arXiv:1912.11211.	The study examined the YouTubes recommendation system in the context of suggesting radicalised content.	2019	22

The Extreme Right Filter Bubble	O'Callaghan, D., Greene, D., Conway, M., Carthy, J., & Cunningham, P. (2013). The extreme right filter bubble. arXiv preprint arXiv:1308.6149.	This study developed a categorization suitable for the analysis of extreme right channels found on YouTube	2013	18
A longitudinal analysis of YouTube's promotion of conspiracy videos	Faddoul, M., Chaslot, G., & Farid, H. (2020). A Longitudinal Analysis of YouTube's Promotion of Conspiracy Videos. arXiv preprint arXiv:2003.03318.	Investigated YouTube's watch-next algorithm to obtain a year-long picture of the videos actively promoted by YouTube	2020	13
KidsTube: Detection, characterization and analysis of child unsafe content & promoters on YouTube	Kaushal, R., Saha, S., Bajaj, P., & Kumaraguru, P. (2016, December). KidsTube: Detection, characterization and analysis of child unsafe content & promoters on YouTube. In 2016 14th Annual Conference on Privacy, Security and Trust (PST) (pp. 157-164). IEEE.	This study aimed to determine the accessibility and detect unsafe content for children.	2016	20
Understanding the Incel Community on YouTube	Papadamou, K., Zannettou, S., Blackburn, J., De Cristofaro, E., Stringhini, G., & Sirivianos, M. (2020). Understanding the incel community on youtube. arXiv preprint arXiv:2001.08293.	The study aims to determine whether the YouTube recommender system facilitates pathways to incel-related videos.	2020	9
The homogeneity of right-wing populist and radical content in YouTube recommendations	Röchert, D., Weitzel, M., & Ross, B. (2020, July). The homogeneity of right-wing populist and radical content in YouTube recommendations. In the International Conference on Social Media and Society (pp. 245-254).	This study investigates the YouTube, recommendations system in the context of German political videos.	2020	0

Auditing Radicalization Pathways on YouTube	Ribeiro, M. H., Ottoni, R., West, R., Almeida, V. A., & Meira Jr, W. (2020, January). Auditing radicalization pathways on YouTube. In Proceedings of the 2020 conference on fairness, accountability, and transparency (pp. 131-141).	This study aimed to investigate whether algorithmic recommendations facilitate pathways to extreme content	2020	73
Down the (White) Rabbit Hole: The Extreme Right and Online Recommender Systems	O,Callaghan, D., Greene, D., Conway, M., Carthy, J., & Cunningham, P. (2015). Down the (white) rabbit hole: The extreme right and online recommender systems. Social Science Computer Review, 33(4), 459-478.	This study aimed to determine whether the recommender system facilitates pathways to the extreme right content.	2015	75
Platformed racism: the mediation and circulation of an Australian race-based controversy on Twitter, Facebook and YouTube	Matamoros-Fernandez, A. (2017). Platformed racism: The mediation and circulation of an Australian race-based controversy on Twitter, Facebook and YouTube. Information, Communication & Society, 20(6), 930-946.	This study aimed to analyse racism from an Australian based controversy on social media platforms	2017	133
The implications of venturing down the rabbit whole	Kaiser, J., & Rauchfleisch, A. (2019). The implications of venturing down the rabbit hole. Internet Policy Review, 8(2).	This study aimed to conduct an exploratory analysis of the YouTube recommender system	2019	7

Examining Sentiments and Popularity of Pro- and Anti-Vaccination Videos on YouTube	Song, M. Y. J., & Gruzd, A. (2017, July). Examining sentiments and popularity of pro-and anti-vaccination videos on YouTube. In Proceedings of the 8th international conference on social media & society (pp. 1-8).	This study aimed to investigate pathways to anti-vaccine videos on YouTube	2017	15
Disturbed YouTube for Kids: Characterising and Detecting Inappropriate Videos Targeting Young Children	Papadamou, K., Papasavva, A., Zannettou, S., Blackburn, J., Kourtellis, N., Leontiadis, I., ... & Sirivianos, M. (2020, May). Disturbed YouTube for kids: Characterising and detecting inappropriate videos targeting young children. In Proceedings of the International AAAI Conference on Web and Social Media (Vol. 14, pp. 522-533).	This study aimed to determine the accessibility and detect unsafe content for children.	2020	8
It is just a flu: Assessing the Effect of Watch History on YouTube, Pseudoscientific Video Recommendations	Papadamou, K., Zannettou, S., Blackburn, J., De Cristofaro, E., Stringhini, G., & Sirivianos, M. (2020). " It is just a flu": Assessing the Effect of Watch History on YouTube's Pseudoscientific Video Recommendations. arXiv preprint arXiv:2010.11638.	To assess the availability of pseudoscientific content on YouTube	2020	0
Measuring Misinformation in Video Search Platforms: An Audit Study on YouTube	Hussein, E., Juneja, P., & Mitra, T. (2020). Measuring misinformation in video search platforms: An audit study on YouTube. Proceedings of the ACM on Human-Computer Interaction, 4(CSCW1), 1-27.	To assess the availability of conspiratorial content on YouTube	2020	11

Riding the Wave of Misclassification: How We End up with Extreme YouTube Content	St√∂cker, C., & Preuss, M. (2020, July). Riding the Wave of Misclassification: How We End up with Extreme YouTube Content. In the International Conference on Human-Computer Interaction (pp. 359-375). Springer, Cham.	To assess whether the recommender system facilitates contextually inappropriate content	2020	2
How YouTube Leads Privacy-Seeking Users Away from Reliable Information	Spinelli, L., & Crovella, M. (2020, July). How YouTube Leads Privacy-Seeking Users Away from Reliable Information. In Adjunct Publication of the 28th ACM Conference on User Modelling, Adaptation and Personalization (pp. 244-251).	To Assess the accessibility of unreliable information	2020	0
Why is YouTube Broadcasting Climate Misinformation to Millions? Climate change misinformation	AVAAZ Report	To Assess the extent of climate change misinformation on YouTube	2020	0
Alternative and Extremist Content on YouTube	Chen, A., Nyhan, B., Reifler, J., Robertson, R., & Wilson, C. (2021). Exposure to Alternative & Extremist Content on YouTube. Anti-Defamation League. Retrieved 16 February 2021, from https://www.adl.org/resources/reports/exposure-to-alternative-extremist-content-on-youtube.	To assess pathways to extremist content via the YouTube Recommender System	2021	0

Birds of a Feather Get Recommended Together: Algorithmic Homophily in YouTube's Channel Recommendations in the United States and Germany	Kaiser, J., & Rauchfleisch, A. (2020). Birds of a Feather Get Recommended Together: Algorithmic Homophily in YouTube's Channel Recommendations in the United States and Germany. Social Media+ Society, 6(4), 2056305120969914.	To assess the formation of extremist communities on YouTube	2020	1
Evaluating the scale, growth, and origins of right-wing echo chambers on YouTube	Hosseinmardi, H., Ghasemian, A., Clauset, A., Rothschild, D. M., Mobius, M., & Watts, D. J. (2020). Evaluating the scale, growth, and origins of right-wing echo chambers on YouTube. arXiv preprint arXiv:2011.12843.		2020	1

Supplementary Materials S2: Methods Table

Citation	Type of Problematic Content	Initial Search Queries	Number of Videos/ Channels	Analysis	Classification of Problematic Content
Alfano, M., Fard, A. E., Carter, J. A., Clutton, P., & Klein, C. (2020)	Conspiracy Theories	Martial-Arts<br>Fitness<br>Firearms and other weapons<br>Natural Foods<br>Tiny Houses<br>Gurus	Retrieved 117150 600 video (100 per topic) included in analysis	Web-crawler simulates user moving through the recommendation system to a crawl depth of 5, all the recommended videos were noted at each stage	Manually coded the 100 most-recommended clips from each topic and used a three-point scale, indicating the extent of conspiratorial claims. These were:1 -no conspiracy theories<br>2 -contained some claim that powerful forces influence (or try to influence) society 3 - included several ideas that some groups or individuals influence (or attempt to control) society and they manipulate information about their actions or existence.

Abul-Fottouh, D., Song, M. Y., & Gruzd, A. (2020).	Pro and Anti-Vaccine Related Videos	Immuni* OR vaccin* OR vaxx*	Retrieved 8425 videos. After exclusion dataset consisted of 2121 videos	Social Network Analysis - LOLOG Model (describes probability distribution over network configurations). Dependent variable - the probability of tie formation among videos in the recommendation network. Modelled the network, tendency to create ties by adding the video, sentiment towards vaccination as the model, primary independent variable. Anti-vaccine videos were the baseline. Another independent variable was whether videos were connected by a tie share from the same sentiment. Also checked for if videos reciprocally recommended each other.	Public health experts watched all vaccine-related videos and categorized them as pro-vaccine (supported immunization), anti-vaccine (refusal attitude or rejection of vaccines) or neutral sentiment (news media presenting both sides of "debate".
Schmitt, J. B., Rieger, D., Rutkowski, O., & Ernst, J. (2018).	Extremist content embedded in networks produced from counter message campaign search terms	Two networks built from two counter-message search queries. Seed videos picked from search queries. (#WhatIS = eight videos; ExitUSA = four videos)	#WhatIS = 185s7 analysed videos. ExitUSA = 980 analysed videos.	Social Network Analysis - Using GEPHI (Crawl Depth = 2). Scraped the recommender system from seed videos and created a nodes and edges list from GEPHI. Conducted a modularity analysis (quality of clustering) to determine communities of videos. Assigned Eigenvector centrality to each node (node) - determines the number of connections a node has to other nodes, the higher the number, the higher its importance to the network.	Produced a randomised sample - 30% of videos in each community and then classified them by their YouTube tags and then manual coders watched the videos to check for extremist/problematic content. Problematic/extremist content regarded as conspiratorial content, hate speech, Islam-related propaganda, and right-wing extreme content.

Ledwich, M., & Zaitsev, A. (2019).	Radicalised Content	Criteria: Channel has over ten thousand subscribers. More than 30 percent of the content on the channel is political.	816 channels	Conducted a social network analysis and estimated the number of times a video was recommended to a user (impressions). Assessed the number of impressions a video was receiving.	Channels were tagged. The soft tags were: conspiracy, libertarian, anti-SJW, social justice, white identitarian, educational, late-night talk shows, partisan left, partisan right, anti-theist, religious conservative, socialist, revolutionary, provocateur, Men Rights Activists, Missing Link Media, state-funded, anti-whiteness. Three labellers allocated tags to each channel. If two or more labellers defined a channel by the same label, that label was assigned to the channel. An intraclass coefficient was used to determine the agreement amongst labellers. Assembled thirteen aggregate groups that represented the political perspectives of the included channels.
O'Callaghan, D., Greene, D., Conway, M., Carthy, J., & Cunningham, P. (2013).	Extreme Right Content	Two data sets associated with extreme-right English language and German language Twitter accounts were generated, by retrieving profile data over an extended period.	English Channels 26,460 and 3,046 German	1. Aggregation process to rank seed channels. 2. Generated TF-IDF channel document vectors and identified topics using NMF. 3. Categorised the classified topics concerning the set defined in Table 1. 4. Categorised the channels based on their topic weights in H. 5. Investigate whether an extreme right filter bubble was present.	Topic Modelling - channels categorized into the following topics: Anti-Islam, Anti-Semitic, Conspiracy Theory, Music, Neo-Nazi, Patriot, Political Party, Populist, Revisionist, Street Movement, White Nationalist.

Faddoul, M., Chaslot, G., & Farid, H. (2020).	Conspiracy Theories	Most Subscribed English Channels	1080 videos	Longitudinal Design (15 months, daily recommendations). 8 million recommendations from YouTube watch-next algorithm. Used a cluster analysis to identify a set of popular US based news channels (1000 recent videos from these channels were the seed videos). Collected recommendations from this set of videos and then measured the proportion of recommended conspiratorial content.	Training Set of conspiratorial videos criteria: Describes situations as covert plots by powerful forces while disregarding accidents, dismissive of scientific consensus, and content not backed by facts and unfalsifiable. Binary classifier identified conspiratorial content. Used the videos title, description, tags, transcript, and comments. The classifier labelled the chance of content including conspiratorial content – scored between 0 (minimal likelihood) and 1 (maximal likelihood).
Kaushal, R., Saha, S., Bajaj, P., & Kumaraguru, P. (2016, December)	Unsafe content for Kids	Top 20 popular cartoon keywords (mickey mouse, tom and jerry, etc.)	408 Seed Videos 262 nodes and 630 edges	Social Network Analysis. They created a directed graph of related video network videos as nodes and edges as recommendations. The algorithm took top 10 recommendations from each video in the seed dataset. If the related video is from among the seed videos, an edge was counted. Counted potential pathways (edges) between safe and unsafe videos in the network. Modularity analysis was also used to detect communities of videos.	Classifier Model (80:20 training to test split) tested on 50 latest videos uploaded for each unique uploader. The aim was to determine whether videos were decent or indecent for children. The classifier model was based on the video, user, and comments to determine if a video is safe or unsafe.

Papadamou, K., Zannettou, S., Blackburn, J., De Cristofaro, E., Stringhini, G., & Sirivianos, M. (2020).	Incel-Related Videos	19 incel-related subreddits searched for YouTube links	1773 incel related videos and 16521 other videos	Top 10 recommended videos for incel related videos and control sets collected from YouTube API. The researchers built a directed graph with nodes (videos) and edges (recommendations). Measured the prevalence of incel related videos in the network. The researchers Calculated the out-degree in terms of incel-related and Other labelled nodes. To measure if YouTube facilitates incel-related communities, the researchers use a random walker (crawl depth = 5 repeated 1000 times). The start of the walk either starts from an incel related video or an unrelated video. Random walker analysed likelihood of encountering incel content, measured % of incel related video encounter on walks.	They built a lexicon of commonly used words by the incel community (200 terms). Researchers identified incel related terms and included them in the lexicon if they indicated hate, misogyny, or is directly associated with incel ideology. Used these terms to decide if a video was incel related. Annotators looked through video transcripts, titles, tags, and comments. Classified as Incel related if: one incel-related term in the video and three in the comments (based on F1 scores).

Rochert, D., Weitzel, M., & Ross, B. (2020, July).	Extremist Content	Two datasets – right-wing populist and politically neutral videos	1,663 German political videos	Social Network Analysis - random walk algorithm. The random walk starts from a video from the list of ten initial videos. Then randomly clicks on a recommended video. Then the random walker selects another video from the recommender system. Store the sequence of nodes and their attributes for each run. This process is repeated 5000 times until the videos from the initial dataset have been passed. To measure video content homogeneity, the researchers used the E-I index (identifies the direct links from nodes to their recommendations based on the class). Values are based on -1 to 1 (-1 = heterogeneous network, 1 = homogeneous network).	Coding Scheme for Right-wing populist or radical content Polemical and polarizing speech, assumptions based without evidence (e.g. Protagonist shows xenophobia and misogyny) hostility towards out-groups, political opponents (esp. the left) (e.g. right-wing extremists, Nazi symbols in the video), conspiracy theories and climate change denial. Neutral Politically unbiased reporting including documentaries or reports on political issues, or political bias in reporting, Protagonist takes a neutral position and shows both sides of the argument. Others Content without political reference such as blogs, vlogs, beauty, lifestyle and gameplay Left-wing content
Ribeiro, M. H., Ottoni, R., West, R., Almeida, V. A., & Meira Jr, W. (2020, January).	Radicalised Content	Media, the Alt-lite, the Intellectual DarkWeb and the Alt-right.	330,925 videos posted on 349 channels	Social Network Analysis of YouTube's recommendation system (nodes represent videos and edges represent recommendations) - conducted simulations on recommendation graphs. Random walks (5 steps) used to determine the reachability of problematic content from random start points.	Seeds were independently annotated twice and removed if disagreement. Set of keywords for each community (alt-lite, alt-right, I.D.W). Search each keyword on YouTube (first 200 results) and add channels related to each community.

O'Callaghan, D., Greene, D., Conway, M., Carthy, J., & Cunningham, P. (2015).	Extreme Right Content	URLs for YouTube from Extreme Right Twitter Accounts	English Language = 26,460 and German Language = 3,046	Seed channels assigned an aggregated ranking by related channels. Identification of topics using topic modelling. Checked the amount of extreme right channels related to each seed extreme right channel.	To classify content, researchers used metadata of videos(titles, descriptions and associated keywords. Channels were also categorized using a schema of keywords based on Extreme right research: Anti-Islam, Anti-Semitic, Conspiracy Theory, Music, Neo-Nazi, Patriot, Political Party, Populist, Revisionist, Street Movement, White Nationalist.
Matamoros-Fernandez, A. (2017).	Racism based Australian Controversy involving Adam Goodes	Tweets containing URLS to YouTube Videos about the Adam Goodes Controversy	3 Seed Videos	Social Network Analysis - video networks based on YouTube recommended videos algorithm and using the YouTube Data Tools	Open coded 529 YouTube links from Twitter
Kaiser, J., & Rauchfleisch, A. (2019).	Indecent Videos of Children and Sexually suggestive Content	political channels, conspiracy theory channels, and the top 250 channels from SocialBlade.com	Starting seed list of 1,851 Resulted in a network of 12,341 channels	Social Network Analysis - Top 20 recommended videos of channels containing sexually suggestive images of underaged women. Utilised modularity analysis (community detection) to understand the channel network.	Sexually suggestive videos containing underaged women

Song, M. Y. J., & Gruzd, A. (2017, July).	Anti-Vaccine Content	"Vaccines" and its derivatives	250 Seed Videos, 1,984 videos directly related to vaccination	Social Network Analysis - assigned network centrality measures to each video - betweenness centrality, in-closeness centrality, out closeness centrality, in-degree centrality, and out-degree centrality. Determine the extent to which a video influences the network. They then conducted a t-test to assess the variance of centrality between pro-vaccine and anti-vaccine videos.	Author with a public health background watched 1,984 vaccine-related videos and categorised them by sentiment (pro-vaccine, anti-vaccine, and neutral).

Papadamou, K., Papasavva, A., Zannettou, S., Blackburn, J., Kourtellis, N., Leontiadis, I., ... & Sirivianos, M. (2020, May).	Inappropriate Videos Targeting Kids	List of 64 keywords - extracted n-grams from the title of videos posted on /r/ElsaGat. List of 83 keywords - extracted n-grams from the title of videos posted on /r/fullcartoonsonyoutube. Random YouTube video identifiers downloaded from API. Collected the most popular videos in the USA, the UK, Russia, India, and Canada, between November 18 and November 21, 2018,	12K seed videos and 844K videos recommended from the seed videos.	Conducted a binary classifier to detect how many videos were inappropriate or appropriate for children in the dataset created a directed graph with nodes as videos and edges as recommendations between videos. Checked the number of transitions from appropriate to inappropriate videos and vice versa. Conducted random walks to determine the likelihood of reaching inappropriate content from appropriate content. Random walks were set for ten steps through the recommender system, and each video was classified along the way (repeated 100 times)	Created ground truth dataset and then used a deep learning model to detect disturbing videos for children. Manual annotation process: Suitable: Appropriate for children aged 1-5 and relevant to specific age group interest. Disturbing: Sexually suggestive scenes and language, child abuse, and horror. Restricted: Not intended for children - rated R. Irrelevant: Appropriate content but not related to a child's interest.

Papadamou, K., Zannettou, S., Blackburn, J., De Cristofaro, E., Stringhini, G., & Sirivianos, M. (2020).	Pseudoscientific Content	Four topics: COVID-19, the anti-vaccine movement,, and the flat earth theory	6.6K unique videos - (1.1K seed videos and 5.5K videos recommended from seed videos).	Crowdsourced Annotators (992) each video presented to three annotators and they decide to label the videos as science, pseudoscience, ethics. Science - content relates to the systematic study of the natural world Pseudoscience - rejects scientific consensus, unfalsifiable, ideas without grounds in scientific methods, explains events as secret plots by powerful forces rather than overt activities or accidents. The classifier then uses snippets, video tags, transcript, and the top 200 comments of a video to detect videos containing pseudoscientific and scientific content.	Crowdsourced Annotators (992) each video presented to three annotators and they decide to label the videos as science, pseudoscience, ethics. Science - content relates to the systematic study of the natural world Pseudoscience - rejects scientific consensus, unfalsifiable, ideas without grounds in scientific methods, explains events as secret plots by powerful forces rather than overt activities or accidents. The classifier then uses snippets, video tags, transcript, and the top 200 comments of a video to detect videos containing pseudoscientific and scientific content.

Hussein, E., Juneja, P., & Mitra, T. (2020).	Conspiracy Theories	Search Topic: 9/11 Conspiracy Theories, Chemtrail Conspiracy Theory, Flat Earth, Moon landing Conspiracy Theories, Vaccine Controversies. Sample Search Query: 9/11 inside job, 9/11 tribute, 9/11 conspiracy, chemtrail, chemtrail flu, chemtrail pilot, flat earth proof, is the earth flat, moon, moon hoax, moon landing china, anti-vaccine, vaccines, vaccines revealed	56,475 videos with 2,943 unique videos	Analysed the following factors: search results (top 20 results), the up-next video recommendation, and the top 5 related videos. Accounted for user personalization by using bots and creating google accounts.	Each video was given an annotation value: -1 debunking, mocking, disproving related misinformation. 0 - neutral & related to misinformation. 1 - promoting, supporting, justifying, explaining related misinformation. 2 - debunking, mocking, disproving unrelated misinformation. 3 - neutral & related to another misinformation. 4 - promoting, supporting, justifying, explaining unrelated. 5 - not about misinformation. 6 - a foreign language. 7 - undefined/unknown. 8 - removed. Then, a scoring metric was used called the SERP-MS (SERP Misinformation Score) to determine the amount of misinformation in a video and its ranking in the search results.

Stocker, C., & Preuss, M. (2020, July).	Contextually Inappropriate Content		10000 videos and 1000 users	The simulation system's basis is a coordinate system modelled after the initial system's assumed latent space. Utilised a 256-dimensional unit hypercube; videos were coordinated by their "real" position, following its properties. Also, an apparent position of a video was recorded (the real position with noise). Collected couples of videos that were seen after each other by users and applied a weak force between them in the apparent space. They calculated the expected distance between two random points in a unit hypercube.	Distance and position of videos in the simulation used to determine if the videos are contextually related. If the videos are distant, then they are contextually unrelation and if they are close together, they might be contextually related. Contextually inappropriate content in the context is defined as a video that violates the viewer's assumptions, intentions, and goals or the uploader of a specific video in the context of the current viewing session.
Spinelli, L., & Crovella, M. (2020, July).	Misinformatio n	Top 10 News Google Searches of 2017 in the United States, Hurricane Irma; Las Vegas shooting; Solar Eclipse; Hurricane Harvey; Bitcoin Price; North Korea; Hurricane Jose; Hurricane Maria; April the Giraffe; and DACA.	25,091 unique videos	Python - Selenium to simulate user behaviour. Created a data collection structure that represents users watching videos on YouTube in different contexts(differing privacy settings and video selection procedures). Classified recommended channels and tracked changes over time.	Trustable: established news sources. Extreme: Denial of scientific knowledge, incite hate and promote fake news. Neutral: neither trustworthy nor extreme.

AVAAZ Report	Climate Change Misinformation	Global warming, climate change and climate manipulation.	5,537 videos	Social Network Analysis - using GEPHI. identified a list of the 100 most-recommended videos for each search term	Manual labelling method - defined climate denial and misinformation as verifiably false or misleading information assessed against the scientific consensus which could adversely impact public safety, by reducing public support for initiatives to reduce human-induced climate change. Fact checked claims using reputable outlets and scientific journals and also checked the source credibility of claims.
Chen, A., Nyhan, B., Reifler, J., Robertson, R., & Wilson, C. (2021).	Alternative Influence Network and Extremist Content	Used browser plugin to track the videos participants watched, the algorithmic YouTube recommendations, and their visits to YouTube	206 alternative and 53 channels viewed by one or more participant and 6 channels that were viewed but eventually taken down	Used browser plug in information to determine what videos participants actually watched in relation to the videos they were recommended. Used data from before installation and data collected by the plug in. Also used representative sample to determine participants' prior attitudes (such as racial resentment)	Alternative Influence Network defined by data society report, alt-lite, and Intellectual Dark Web identified by Ribeiro et al, and the anti-SJW and Mens rights categories identified by Ledwich and Zaitsev. Extremist channels identified as white supremacist, alt-right as identified by Ribeiro et al and white identiterian as identified by Ledwich and Zaitsev or as extremist/hate content.

Kaiser, J., & Rauchfleisch, A. (2020). Birds of a Feather Get Recommended Together: Algorithmic Homophily in YouTube's Channel Recommendations in the United States and Germany. Social Media+ Society, 6(4), 2056305120969914.	Extremist Content	A list of far-right and far-left channels and a list of 250 news mainstream channel	13529 channels in the United States and 8000 in Germany	Conducted a social network analysis, using GEPHI. They ran a modularity analysis to identify the distinctiveness of communities within the networks.	Classified communities by the most common type of channels that they contained. Read the titles and channel descriptions. Used language analyzer to determine political themes within communities.

Hosseinmardi, H., Ghasemian, A., Clauset, A., Rothschild, D. M., Mobius, M., & Watts, D. J. (2020). Evaluating the scale, growth, and origins of right-wing echo chambers on YouTube. arXiv preprint arXiv:2011.12843.	Far-right extremist content	Panellist data of 309, 813 panellists (logged one youtube view). Over 4 year period	Number of unique users 309, 813 Number of watched-video pageviews 21, 385, 962 Number of unique video IDs 9, 863, 0964 Number of unique channel IDs 2, 293, 760 Number of sessions 8, 620, 394	Identified consecutive page views by a user, and assigned users to clusters of videos that are highly related to each other. To determine overall trends to radical content, overall changes in total consumption associated with each of the content communities was investigated	far left (fL), left (L), centre (C), right (R) and far right (fR) - Classifications of videos based on the political leaning of the channel

Supplementary Materials S3: Results Table

**Title**	**Results/Findings**	**Does The Recommender System Facilitate Pathways To Problematic Content?**
Technologically scaffolded atypical cognition: the case of YouTube's recommender system	YouTube recommender system promoted conspiracy theories from all search queries.<br>The following order of content represents the amount conspiratorial content found in each category:<br>gurus > natural foods > firearms > fitness > martial arts > tiny houses	Yes
Examining algorithmic biases in YouTube's recommendations of vaccine videos	YouTube recommended more neutral and pro-vaccine videos than anti-vaccine videos. The study detected a homophily effect. Pro-vaccine videos recommended further pro-vaccine videos and anti-vaccine vids recommended further anti-vaccine content. Anti-vaccine videos were less likely to facilitate pathways towards pro-vaccine videos.	Mixed Results

Counter-messages as Prevention or Promotion of Extremism?! The Potential Role of YouTube: Recommendation Algorithms	The study showed that Counter Message Videos are close or directly related to extremist content.	Yes
Algorithmic Extremism: Examining YouTube Rabbit Hole of Radicalization	Recommender system did not facilitate pathways to radicalisation or extremist content. The recommender system favoured mainstream media and politically neutral channels as opposed to problematic content.	No
The Extreme Right Filter Bubble	The results found a filter bubble. YouTube Users who watch an extreme right video are highly likely to be recommended other extreme right content.	Yes

A longitudinal analysis of YouTube's promotion of conspiracy videos	No substantial evidence for pathways or push towards right-wing political content. High chance of conspiratorial video recommendation after watching conspiracy video, this likelihood is decreasing.	Mixed Results
KidsTube: Detection, characterization and analysis of child unsafe content & promoters on YouTube	safe to unsafe transitions were uncommon. The unsafe to unsafe pathways were more common; occurring 117 times.	Mixed Results
Understanding the Incel Community on YouTube	6% chance of randomly encountering incel-related video from "other" video via recommender system. 18.8 % chance of meeting incel-related video from a non-related video within five hops via the recommender system.	Yes

The homogeneity of right-wing populist and radical content in YouTube recommendations	The recommendation network demonstrated a high degree of homogeneity of right-wing populist and politically neutral videos.	Yes
Auditing Radicalization Pathways on YouTube	The recommender system facilitated suggestions to Alt-lite and Intellectual Dark Web Content. The study found pathways via the channel recommender system from Alt-lite and Intellectual Dark Web Content to Alt-right content, but not via recommended videos.	Mixed Results
Down the (White) Rabbit Hole: The Extreme Right and Online Recommender Systems	More extreme right videos are accessible via the recommender system after watching an extreme right video. The authors introduce the concept of an 'ideological bubble' after just a few clicks.	Yes

Platformed racism: the mediation and circulation of an Australian race-based controversy on Twitter, Facebook and YouTube	The recommender system facilitated controversial humour about the Adam Goodes racism topic and videos by public figures who have shared racist remarks about Aboriginal people.	Yes
The implications of venturing down the rabbit whole	Communities of sexually suggestive channels and some of these channels contained indecent videos of children. 50% of these videos were reachable via the recommender system from the seed channels. However, most were ten jumps away. Videos were accidentally identified while analysing Brazilian political videos.	Mixed Results

Examining Sentiments and Popularity of Pro- and Anti-Vaccination Videos on YouTube	anti-vaccine videos were more important to the network, per closeness centrality. The analysis showed anti-vaccine content facilitates pathways to more anti-vaccine videos on YouTube.	Yes
Disturbed YouTube for Kids: Characterising and Detecting Inappropriate Videos Targeting Young Children	The authors conclude that children could be recommended problematic content when they randomly search from benign or appropriate videos.	Yes

It is just a flu: Assessing the Effect of Watch History on YouTube, Pseudoscientific Video Recommendations	Users were more likely to encounter pseudoscientific videos in the platform's search results page than the recommender system or the homepage. There was also a non-negligible amount of pseudoscientific content on the recommender and homepage sections.	Mixed Results
Measuring Misinformation in Video Search Platforms: An Audit Study on YouTube	User watch history affects the amount of misinformation recommended by YouTube. Filter bubble effect identified across all conspiratorial topics. Watching videos with conspiratorial misinformation lead to more misinformative videos.	Yes
Riding the Wave of Misclassification: How We End up with Extreme YouTube Content	Contextually inappropriate recommendations collateral damage instead of the primary purpose of the recommender system. Autoplay is particularly problematic in facilitating contextually inappropriate content.	Mixed Results

How YouTube Leads Privacy-Seeking Users Away from Reliable Information	YouTube recommendations generally moved users from reliable information to video channels containing extreme and unscientific viewpoints. Results also showed that users who had personal information hidden received a larger amount of extreme and unreliable recommendations.	Yes
Why is YouTube Broadcasting Climate Misinformation to Millions? Climate change misinformation	Sixteen per cent of the top 100 videos detected by the YouTube API contained climate change misinformation. Climate change misinformation videos had 21 million views. Over 20% of the views for the top 100 related videos for the search term global warming contained climate misinformation.	Yes

Exposure to Alternative & Extremist Content on YouTube	9.2% of participants viewed an extremist channel video and 22.1% of participants viewed a video from an Alternative Influence Network channel. When participants watched these videos they were more likely to recommend similar videos. 90% of views for both types of videos came from participants who scored highly in racial resentment. Recommendations from other types of video are rare but common when topics align.	Yes
Birds of a Feather Get Recommended Together: Algorithmic Homophily in YouTube's Channel Recommendations in the United States and Germany	Identified the formation of homophilous far-right communities on YouTube - evidence for a filter bubble effect	Yes

Evaluating the scale, growth, and origins of right-wing echo chambers on YouTube	Growing Far-right echo chambers, however, no evidence to support notion that they are caused by the recommendation algorithm	No

Supplementary Materials S4: Classification of Problematic Content Table

Citation	Extremist	Conspiratorial	Anti-Vaccine	Pseudoscientific	Content Unsafe for Children	Incel-Related	Radicalising	Racist
Alfano et al. (2020)		▲						
Hussein et al. (2020)		▲						
Faddoul et al (2020)		▼						
Ledwich & Zaitsev (2019)		○					●	○
Song and Gruzd (2017)			▲					
Abul-Fottouh, Song, and Gruzd (2020)			▼					
Papadamou et al. (2020)			▽	▼				
Spinelli and Crovella (2020)				▲				
AVAAZ (2020)				▲				
Papadamou et al. (2020)					▲			
Kaiser and Rauchfleisch (2019)					▼			

Kaushal, Saha, Bajaj, and Kumaraguru (2016)		▼		
Stöcker & Preuss (2020)		▼		
Papadamou et al. (2020)			▲	
Chen, Nyhan, Reifler, Robertson, & Wilson (2021)	▲			
O’Callaghan, Greene, Conway, Carthy, & Cunningham (2013)	▲			
O’Callaghan, Greene, Conway, Carthy, & Cunningham (2015)	▲			△
Röchert, Weitzel, & Ross (2020)	▲			

Schmitt, Rieger, Rutkowski, & Ernst (2018)	▲			
Kaiser & Rauchfleisch (2020)	▲	△		
Ribeiro et al. (2020)			▼	
Matamoros-Fernández (2017)				▲
Hosseinmardi et al. (2020)	●			

Key: ***Triangle*** *= Recommender system facilitated pathways to problematic content*
Upside down Triangle *= Mixed Results*
Circle *= Recommender system did not facilitate pathways to problematic content*
Black Shading *= Target problematic content*
White Shading *= Proxy*
